# Comparative Impact of Microsurgical Varicocelectomy Versus Observation on Infertility in Infertile Men With Subclinical Varicocele

**DOI:** 10.7759/cureus.77477

**Published:** 2025-01-15

**Authors:** Petros Sountoulides, Nikolaos Pyrgidis, Aris Kaltsas, Stavros Gravas, Dimitrios Kikidakis, Ioannis Zachos, Athanasios Zachariou, Fotios Dimitriadis, Nikolaos Sofikitis

**Affiliations:** 1 Department of Urology, General Hospital of Veroia, Veroia, GRC; 2 Department of Urology, Ludwig-Maximilian University Hospital, Munich, DEU; 3 Department of Urology, University of Ioannina, Ioannina, GRC; 4 Department of Urology, University of Thessaly, Larissa, GRC

**Keywords:** assisted reproductive technology (art), male factor infertility, semen parameters, subclinical varicocele, varicocelectomy

## Abstract

Purpose: The efficacy of varicocelectomy in treating infertility among men with ultrasonography-detected subclinical varicocele continues to be a topic of clinical uncertainty. This multicentric, prospective, non-randomized study aimed to evaluate the effect of microsurgical ligation of the internal spermatic vein in men with left subclinical varicocele and no other infertility causes.

Patients and methods: The study included 34 infertile men diagnosed with subclinical left varicocele by color Doppler ultrasonography. After a shared decision-making process, the participants self-selected either microsurgical ligation (n = 18) or conservative management (n = 16). Baseline age, duration of infertility, reproductive hormone levels, and semen parameters were comparable across groups. The primary outcomes included changes in sperm concentration, progressive motility, and morphology over a nine-month follow-up period.

Results: After nine months, there were no statistically significant differences between the surgery and observation groups in sperm concentration or progressive motility. After adjusting for baseline values, sperm concentration increased by 3 × 10^6^/mL (95% CI: −6.1 to 12.1), and progressive motility improved by 7.1% (95% CI: −1.3 to 15.5%) post-surgery, although these increases were not significant. However, the surgical group demonstrated a significant 5.6% improvement in sperm morphology (95% CI: 0.1 to 11.2%, p = 0.045) compared to observation.

Conclusion: Although this study's small sample size limits its broader generalizability, its findings provide limited evidence that microsurgical ligation may modestly improve sperm morphology in men with subclinical varicocele. Further research with larger, randomized cohorts is warranted to confirm these results and assess potential fertility outcomes.

## Introduction

Male infertility affects a significant proportion of couples worldwide, with varicocele recognized as one of the most common surgically treatable causes. Varicocele, characterized by the abnormal dilation and tortuosity of the pampiniform plexus of the internal spermatic veins, occurs in approximately 15-20% of the general male population and up to 30-40% of men presenting with infertility [1,2]. Although varicocele is the most frequently encountered surgically correctable factor in male infertility, its exact pathophysiological mechanisms remain partly unclear. Proposed mechanisms include increased scrotal temperature, oxidative stress, venous hypertension, and impaired testosterone regulation, all of which can disrupt testicular function and spermatogenesis [3]. Excessive production of reactive oxygen species (ROS) in varicocele has been implicated in DNA damage, lipid peroxidation, and inflammation, further exacerbating testicular injury [4,5].

Surgical correction remains the mainstay of treatment for varicocele-associated infertility, and various techniques, including open, laparoscopic, robotic, and microsurgical varicocelectomy, have been utilized to improve semen parameters and potentially enhance pregnancy outcomes [6,7]. For men with a palpable varicocele and abnormal semen analysis, numerous studies have demonstrated improvements in sperm concentration, motility, or morphology following varicocelectomy [8,9]. In contrast, the benefits of treating subclinical varicocele, defined as a varicocele detectable only by Doppler ultrasound rather than physical examination, are still debated, given the relatively sparse and heterogeneous data regarding its impact on fertility [10]. Indeed, the current European Association of Urology (EAU) and American Urological Association/American Society for Reproductive Medicine (AUA/ASRM) guidelines recommend avoiding surgical intervention for subclinical varicocele due to insufficient evidence [11,12].

Nevertheless, sperm morphology remains a pivotal determinant of male fertility, as structurally abnormal spermatozoa often demonstrate reduced fertilization capacity and decreased pregnancy rates, both naturally and with assisted reproductive technologies (ART) [13]. Given the ongoing debate and the reliance on older or methodologically diverse studies, we conducted a multicentric, prospective study to assess the effects of microsurgical subinguinal varicocelectomy in infertile men diagnosed with left subclinical varicocele, focusing on key semen parameters. This approach allowed us to evaluate whether surgical correction in this often-overlooked subgroup confers any measurable benefits in sperm quality.

## Materials and methods

This multicentric, prospective, observational study was conducted across three urology departments in Greece: the University Hospital of Ioannina (Ioannina), the General Hospital of Veroia (Veroia), and the University Hospital of Thessaly (Larissa). The study protocol was predefined and approved by each participating Institutional Review Board (IRB) (approval dates: Ioannina: 25/06/2002; Veroia: 18/07/2002; Thessaly: 14/08/2002). All procedures followed the ethical standards of the Declaration of Helsinki and adhered to the Strengthening the Reporting of Observational Studies in Epidemiology (STROBE) guidelines [14]. The recruitment period spanned 2006 to 2009, with the final follow-up concluded in 2010.

Although some aspects of clinical management may have evolved since then, the question of how best to treat subclinical varicocele remains a subject of debate, rendering these findings relevant for ongoing discussions and future research. Participants were required to be infertile men aged 18 years or older, with at least one year of unsuccessful attempts at conception through regular, appropriately timed intercourse. All enrolled men had a pathological semen analysis based on the 2010 World Health Organization (WHO) criteria [15] and evidence of left subclinical varicocele on color Doppler ultrasound, defined by a venous diameter exceeding 3 mm and reflux lasting more than two seconds during the Valsalva maneuver in the upright position. Patients were included only if they had normal baseline levels of reproductive hormones (luteinizing hormone, follicle-stimulating hormone, testosterone, prolactin, estradiol), and female factor infertility had been excluded through clinical, laboratory, and imaging studies.

The exclusion criteria comprised congenital or acquired urological conditions (e.g., cryptorchidism, orchitis, orchiectomy, testicular torsion) or any sexual or erectile dysfunction. Patients were also excluded if they had chronic conditions known to affect fertility (e.g., diabetes mellitus, chronic kidney disease, significant endocrinopathies), had used medications or recreational drugs within the last six months that might impair fertility, or had psychiatric illnesses. Prior surgeries or comorbidities posing potential confounders were additional grounds for exclusion, although detailed data on smoking, alcohol consumption, or minor surgeries were not systematically collected.

All participants and their partners were evaluated in a multidisciplinary infertility clinic. After excluding female factor infertility, eligible male patients underwent a thorough assessment that included detailed medical, sexual, and reproductive histories; physical examination; hormone profiling; and high-resolution color Doppler ultrasound of the scrotum. Two semen samples were obtained from each participant, each following a minimum of two days of abstinence, and analyzed within one hour of collection according to WHO standards. A semen analysis was deemed pathological if sperm concentration, progressive motility, or morphology fell below the fifth percentile of the 2010 WHO reference values [15].

Upon confirming eligibility, patients were informed of both surgical and conservative options, including the possible advantages, limitations, and anticipated outcomes of each strategy. Treatment decisions were made through a shared decision-making process, wherein all participants received uniform, structured counseling. This approach minimized bias related to patient preference, and baseline characteristics (e.g., age, fertility history, semen parameters) were carefully documented to permit adjustment for potential confounders in subsequent analyses.

To address potential bias introduced by allowing patients to choose their treatment, the study implemented several strategies to standardize information and minimize variability. All patients received identical information about both treatment options, including the potential benefits, risks, and likely outcomes, in a structured counseling session conducted by trained clinicians. This standardized approach ensured that all participants made informed decisions based on consistent information, regardless of their choice. Additionally, baseline characteristics, including age, fertility history, and semen parameters, were carefully documented to allow for adjustment in the analysis and to control for potential confounding factors related to treatment choice.

**Figure 1:**
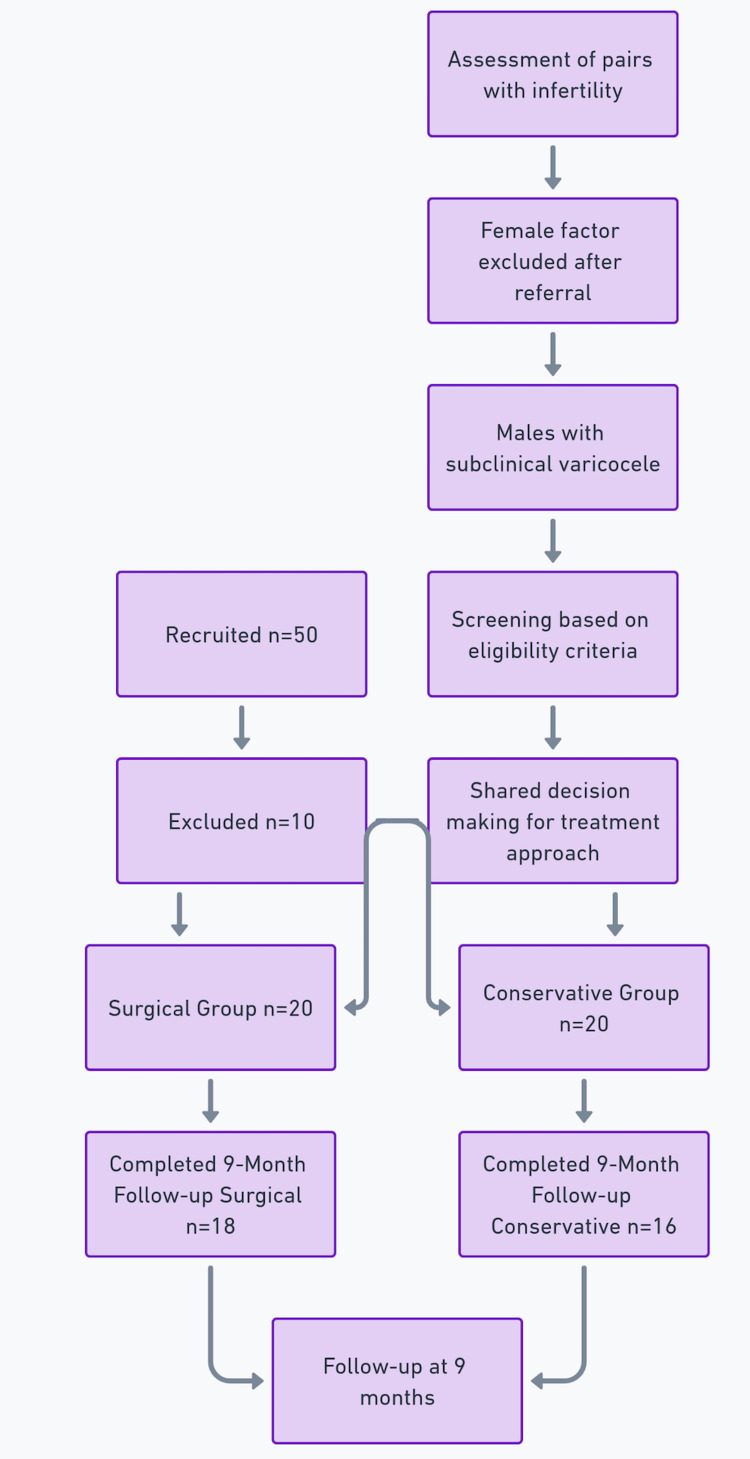
Study flowchart depicting patient enrollment, treatment allocation, and follow-up protocol. Image created by the authors

Those opting for surgery underwent microsurgical subinguinal ligation of the internal spermatic veins under general anesthesia. A small subinguinal incision was used to access and ligate the veins under microscopic visualization, an approach associated with lower recurrence and complication rates compared to other varicocelectomy techniques [6]. All procedures were performed by an experienced urologist, with monitoring for adverse events (such as infection, hydrocele, testicular atrophy, and varicocele recurrence) throughout a nine-month postoperative period. No complications were detected in this series. Participants choosing conservative management were asked to avoid alternative infertility treatments. Both groups underwent follow-up examinations at nine months, which included repeat semen analyses, color Doppler ultrasound, and clinical evaluations (Figure 1).

The primary endpoint was the mean change in semen parameters (sperm concentration, progressive motility, and morphology) from baseline to nine months, comparing surgical and conservative cohorts. Secondary endpoints included the respective effects of surgical or conservative management on semen parameters at follow-up and the occurrence of any adverse events in either group. Descriptive statistics were used to characterize baseline data, with continuous variables reported as medians and interquartile ranges (IQR) for non-normal data or as means and standard errors (SE) for normal data, and categorical variables expressed as frequencies and percentages. Normality was assessed via the Shapiro-Wilk test. The Mann-Whitney U test compared baseline versus follow-up outcomes between groups, and within-group comparisons used the Wilcoxon signed-rank test. Mean changes in semen parameters and 95% confidence intervals (CI) were calculated, and an analysis of covariance (ANCOVA), adjusted for baseline values, was conducted to examine between-group differences. All analyses were performed with R version 3.6.3 (R Foundation for Statistical Computing, Vienna, Austria), and statistical significance was set at p < 0.05. A biostatistician supervised the entire analysis process to ensure accuracy.

Prior to study initiation, a sample size calculation indicated that a minimum total of 26 participants was required to detect a clinically relevant improvement in sperm morphology (effect size 0.8, power 80%, α = 0.05). In total, 34 patients met the strict eligibility criteria for left subclinical varicocele without other causes of infertility, surpassing the initial target. However, due to the inherent rarity of this strictly defined condition, the study may still be underpowered to detect subtler differences in semen outcomes. Therefore, the results should be interpreted with caution.

## Results

A total of 34 consecutive male patients were included in the study, each diagnosed with subclinical left varicocele and no other identifiable causes of infertility. Of these, 18 opted for microsurgical correction, while 16 chose conservative management. The median age of the participants was 32.5 years (IQR: 31-34), and the median body mass index (BMI) was 25 kg/m^2^ (IQR: 24-26). No significant differences were observed at baseline between surgical and conservative groups in terms of age (p = 0.78) or BMI (p = 0.83). All participants were Caucasian, and none presented with comorbidities that could confound semen outcomes. No patients were lost to follow-up; all completed the nine-month post-treatment assessment.

Baseline characteristics, including reproductive hormone levels, were comparable between the two groups (Table 1).

**Table 1:** Baseline characteristics of the study participants. Data are presented as mean ± standard deviation (SD). P-values are calculated using t-tests for continuous variables. FSH: Follicle-Stimulating Hormone; LH: Luteinizing Hormone; TT: Total Testosterone; PRL: Prolactin; E2: Estradiol

Characteristic	Surgical Group (n = 18)	Conservative Group (n = 16)	Total (n = 34)	p-value
Age (years)	32.8 ± 4.2	31.9 ± 3.8	32.4 ± 4.0	0.78
FSH (1.3–19.3 IU/L)	15.4 ± 5.2	16.2 ± 5.8	15.8 ± 5.5	0.64
LH (1.2–8.6 IU/L)	7.4 ± 2.1	7.9 ± 2.3	7.7 ± 2.2	0.53
TT (1.8–7.8 ng/mL)	3.0 ± 0.6	3.1 ± 0.5	3.05 ± 0.55	0.48
PRL (2.6–13.1 ng/mL)	6.8 ± 1.4	6.5 ± 1.7	6.65 ± 1.55	0.72
E2 (73–275 pmol/L)	112.3 ± 22.5	118.4 ± 19.8	115.3 ± 21.2	0.36

In the surgical group, no perioperative or long-term complications occurred, and color Doppler ultrasound at follow-up confirmed the absence of residual varicocele in all operated patients.

Table 2 summarizes the baseline and follow-up semen parameters for both groups.

**Table 2:** Patient characteristics and semen parameters at baseline and follow-up. IQR: Interquartile Range

Parameter	Group	Baseline (Median, IQR)	Follow-up (Median, IQR)	p-value (Within-Group)
Sperm concentration (10^6^/mL)	Surgery	12.8 (6.8–17.8)	15 (8–23)	0.297
	Conservative	12 (5.2–18.2)	12 (6.8–18.2)	0.488
	p-value for between groups	0.74	0.56	-
Progressive sperm motility (%)	Surgery	21.5 (8.2–35)	25 (16–29)	0.453
	Conservative	14 (0–22)	10.5 (0–31.8)	0.146
	p-value for between groups	0.37	0.15	-
Sperm morphology (normal forms, %)	Surgery	26.5 (21.2–37)	28.5 (19.5–42.8)	0.794
	Conservative	20 (8.2–29.5)	16.5 (4.5–26.2)	0.068
	p-value for between groups	0.13	0.013	-

At nine months, the surgical group showed nonsignificant increases in sperm concentration (median increase 4 × 10^6^/mL) and progressive motility (median increase 2%), while sperm morphology improved by a median of 3.5%. The conservative group showed minimal changes in sperm concentration (median increase 2 × 10^6^/mL) and a slight decrease in both progressive motility (median -2%) and sperm morphology (median -1.5%). Although these within-group differences were not statistically significant, there was a noteworthy trend favoring an improvement in morphology in the surgical group compared to the conservative group.

At baseline, there were no statistically significant differences between groups for sperm concentration, motility, or morphology. However, at the nine-month follow-up, a significant between-group difference emerged in sperm morphology (p = 0.013). After adjusting for baseline values, the adjusted mean difference in morphology between the surgical and conservative groups was 5.6% (95% CI: 0.1 to 11.2%, p = 0.045), favoring surgery. Adjusted differences for sperm concentration and motility did not reach statistical significance (p = 0.5 and p = 0.1, respectively).

These findings suggest that while microsurgical varicocelectomy may not significantly improve sperm concentration or motility in cases of subclinical varicocele, it appears to confer a modest but statistically significant improvement in sperm morphology. Given the relatively small sample size and the nine-month follow-up period ending in 2010, these results should be interpreted with caution. Nonetheless, the improvement in morphology, an important determinant of fertilization capacity, could be clinically relevant for some patients. Larger studies with longer follow-up periods and more contemporary clinical protocols may help clarify the role of microsurgical correction in subclinical varicocele.

Table 3 provides a detailed comparison of changes in semen parameters between the two groups, with adjustments for baseline values.

**Table 3:** Adjusted changes from baseline in semen parameters between groups. SE: Standard Error; CI: Confidence Interval

Parameter	Surgery Group (Mean, SE)	Conservative Group (Mean, SE)	Unadjusted Mean Difference (95% CI)	Adjusted Mean Difference (95% CI)	Adjusted Between-Group p-value
Sperm concentration (10^6^/mL)	1.6 (4.5)	1 (1.6)	0.6 (-9.4 to 10.5)	3 (-6.1 to 12.1)	0.5
Progressive sperm motility (%)	3.2 (3.3)	-3.3 (2.7)	6.5 (-2.3 to 15.2)	7.1 (-1.3 to 15.5)	0.1
Sperm morphology (normal forms, %)	1 (2.1)	-3.3 (1.6)	4.3 (-1.1 to 9.6)	5.6 (0.1 to 11.2)	0.045

## Discussion

This multicentric, prospective study conducted via shared decision-making suggests that microsurgical correction of an ultrasonography-detected left subclinical varicocele leads to a modest but statistically significant improvement in sperm morphology in infertile males with oligoasthenoteratozoospermia and no other identifiable cause of infertility. At nine months, no significant differences were observed in sperm concentration or progressive motility between the surgical and conservative management groups, but the notable improvement in sperm morphology raises the question of whether surgical intervention can offer meaningful clinical benefits for these patients.

Sperm morphology is a critical determinant of fertilization potential [13], and abnormal morphology is linked to lower natural conception rates [16]. Thus, the 5.6% improvement in sperm morphology seen in our surgical group may translate into a higher probability of conception, although absolute morphology values still vary among individuals [15]. Even modest gains may hold clinical significance for some couples, particularly in the context of ART, where improved morphology has been correlated with better outcomes [17,18].

Despite these encouraging findings, we did not collect data on either natural or assisted pregnancy rates, which is a significant limitation. Pregnancy outcomes are the most relevant measure in fertility research [19], and the absence of such data means we cannot ascertain whether the noted improvements in morphology would ultimately enhance live birth rates.

Several mechanisms could explain why varicocelectomy might improve sperm morphology. Chronic oxidative stress due to elevated scrotal temperature and venous reflux in varicocele has been implicated in DNA damage and apoptosis [4,20]. Surgical correction reduces venous stasis and can improve testicular temperature regulation [21], potentially lowering ROS and restoring healthier spermatogenesis [22]. Additionally, varicocelectomy may normalize endocrine function, as testosterone production can be impaired in varicocele [23,24]. Correction of venous reflux might also alleviate hypoxia-induced injury to the seminiferous epithelium [25,26].

Subclinical varicocele remains an area of ongoing debate, and current guidelines from the American Urological Association/American Society for Reproductive Medicine and the European Association of Urology do not recommend routine surgical intervention [11,12]. Subclinical lesions are often more difficult to diagnose consistently, and study findings vary [7,27]. Some reports indicate only minor improvements after surgery [28], while others show more substantial gains, potentially influenced by differing levels of underlying infertility severity [29]. Moreover, the type of varicocelectomy technique, whether inguinal, subinguinal, or testicular vein embolization, does not appear to drastically alter outcomes in subclinical varicocele [30].

Our study's strengths include its prospective, multicentric design and strict eligibility criteria, focusing on men with subclinical varicocele and excluding other infertility factors. Nevertheless, several limitations should be considered. First, patient self-selection of treatment through shared decision-making may introduce selection bias, as those opting for surgery might differ in motivation or expectations. Second, we did not evaluate pregnancy rates, limiting our ability to draw conclusions about clinical fertility outcomes. Third, there is a substantial time gap between data collection (ending in 2010) and publication, during which clinical practices and diagnostic criteria for varicocele could have evolved. Finally, although our sample size exceeded the initial calculation, the rarity of strictly defined subclinical varicocele may have constrained our ability to detect smaller yet clinically meaningful differences.

Future research should prioritize randomized controlled trials that include pregnancy data to elucidate whether improvements in sperm morphology translate into better reproductive outcomes. Larger cohorts and extended follow-up periods would provide more robust evidence regarding varicocelectomy's role in managing subclinical varicocele under contemporary clinical standards.

## Conclusions

Our study indicates that microsurgical ligation of the left internal spermatic vein yields a modest yet statistically significant improvement in sperm morphology for infertile men with subclinical varicocele and no other identifiable infertility causes. In contrast, no significant changes were observed in sperm concentration or motility compared to conservative management. Given the limited sample size and absence of pregnancy outcome data, it remains unclear whether this improvement in morphology alone justifies surgery over proceeding directly to ART. These findings should, therefore, be interpreted with caution. Larger, more contemporary studies measuring both semen parameters and fertility outcomes, natural or ART-related, are needed to clarify the clinical benefit of surgical correction in this patient population.
